# Bile acids as germinants for *Clostridioides difficile* spores, evidence of adaptation to the gut?

**DOI:** 10.1093/femsre/fuaf005

**Published:** 2025-02-08

**Authors:** Gianni Vinay, Jurgen Seppen, Peter Setlow, Stanley Brul

**Affiliations:** Tytgat Institute for Liver and Intestinal Research, Amsterdam University Medical Centers, Location AMC, University of Amsterdam, Amsterdam 1105 AZ, The Netherlands; Tytgat Institute for Liver and Intestinal Research, Amsterdam University Medical Centers, Location AMC, University of Amsterdam, Amsterdam 1105 AZ, The Netherlands; Department of Molecular Biology and Biophysics, UConn Health, Farmington, CT 06030-3305, United States; Department of Molecular Biology and Microbial Food Safety, Swammerdam Institute for Life Sciences, University of Amsterdam, Amsterdam 1098 XH, The Netherlands

**Keywords:** bacterial spores, Clostridium, germinants, bile acids, germinant receptors

## Abstract

Bacterial spores formed upon metabolic stress have minimal metabolic activity and can remain dormant for years. Nevertheless, they can sense the environment and germinate quickly upon exposure to various germinants. Germinated spores can then outgrow into vegetative cells. Germination of spores of some anaerobes, especially *Clostridioides difficile*, is triggered by cholic acid and taurocholic acid. Elevated levels of these bile acids are thought to correlate with a perturbed gut microbiome, which cannot efficiently convert primary bile acids into secondary bile acids. That bile acids are germination-triggers suggests these bacteria have a life cycle taking place partially in the mammalian digestive tract where bile acids are plentiful; notably bile acids can be made by all vertebrates. Thus, spores survive in the environment until taken up by a host where they encounter an environment suitable for germination and then proliferate in the largely anaerobic large intestine; some ultimately sporulate there, regenerating environmentally resistant spores in the *C. difficile* life cycle. This review summarizes current literature on the effects of bile acids and their metabolites on spore germination in the gut and evidence that adaptation to bile acids as germinants is a consequence of a life cycle both inside and outside the digestive tract.

## Teaser

The review summarizes current literature on effects of bile acids and their metabolites on clostridial spore germinant receptor proteins, their structural organization in germinosomes in spore membranes, and the occurrence of anaerobic spore forming bacteria in the gut together with the evidence that adaptation to bile acids as germinants is a consequence of a life cycle both inside and outside the digestive tract.

## Introduction

*Clostridioides difficile* is an obligate anaerobic, Gram-positive, spore-forming bacterium that is a major cause of antibiotic-associated diarrhea. *Clostridioides difficile* infection (CDI) symptoms range from mild diarrhea and abdominal distress to life-threatening pseudomembranous colitis. Such infection is thought to be potentiated by antibiotic usage, which causes gut dysbiosis by altering the gut microbiota, sensitizing individuals to *C. difficile* colonization. A key property of *C. difficile* is its ability to form spores, as they are critical for the infection cycle and resistant to antibiotics, environmental insults, oxygen, and disinfectants. For *C. difficile* to survive well outside the host, it must form spores, and these spores allow the spreading of *C. difficile* through patient-to-patient contact and environmental exposure. The spores then survive transit through the acidic stomach unharmed, and when they enter the anaerobic gastrointestinal tract, the spores can germinate and outgrow, generating vegetative cells. There are generally significant levels of *C. difficile* spore germination promoting bile acids (BAs) in the intestine. Note that several types of BAs, especially secondary BAs, are quite toxic for *C. difficile* vegetative cells, as will be discussed extensively further on. Subsequently, upon outgrowth and cell division, vegetative cells in the population can begin to produce toxins (TcdA, TcdB, and CDT) (Chandrasekaran and Lacy 2017, Ransom et al. 2018, Kordus et al. 2022) which can cause symptoms. These can range from asymptomatic, to mild diarrhea, and more severe and life-threatening presentations like pseudomembranous colitis, toxic megacolon, dehydration, abdominal distention, hypoalbuminemia with peripheral edema and subsequent circulatory shock (Czepiel et al. 2019). Due not only to the antibiotic resistance and prevalence of the spores, but also the fact that *C. difficile* can cause life-threatening diarrhea which occurs most often in people who have taken antibiotics for other conditions and is the most common healthcare-associated infection, the Centers for Disease Control and Prevention has listed *C. difficile* ‘an urgent threat’ (CDC 2019). Study of the molecular details of *C. difficile* sporulation and spore germination could lead to the development of better prevention methods as well as new compounds for disease treatment and *C. difficile* detection (Abhyankar et al. 2019).

## Spore structure

Many bacterial species in the Bacillota phylum (Firmicutes), specifically the *Bacillales* and *Clostridiales* orders with both aerobic and anaerobic species, have the ability to sporulate. Sporulation in *C. difficile* is a pathway that culminates in the production of a dormant spore, allowing it to persist in the host and disseminate through patient-to-patient contact and the environment. Most is known structurally and mechanistically about Bacillus spores, the reason why we discuss their overall spore structure first and link it next to specific features of *C. difficle*. Clearly, in evolutionary terms the *Clostridiales* preceded the *Bacillales*.

In contrast to vegetative cells, spores have little if any metabolic activity and are very resistant to harsh conditions such as elevated temperatures, radiation, desiccation, oxygen, antibiotics, and other chemicals (Setlow 2014). Spores are among the most resilient cell types known and can survive for long periods of time; it has been claimed that viable spores have been isolated from the gut of extinct bees buried in amber more than 25 million years ago (Cano and Borucki 1995). However, there may be limits on spore longevity due to aspartate isomerization, which can lead to damage to proteins (Liang et al. 2019), as well as effects of background γ-radiation that can damage DNA (Horne et al. 2022).

**Figure 1.**
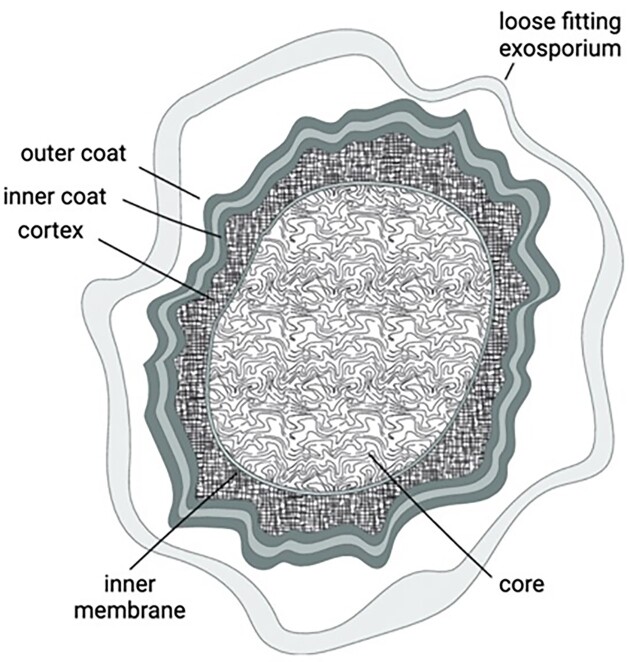
Overview of the common ultra-structure shared by endospores of Bacillales and Clostridiales. Some species, such as *C. difficile*, have a large loose fitting exosporium, as depicted in Fig. 1 (Setlow 2019), others, such as *B. subtilis*, present a crust (Christie and Setlow 2020).

Resistance of the spores is largely due to their ultrastructure, which is similar in different spore formers (Fig. 1). From the outside, common spore layers include an outer coat that protects inner layers against reactive chemicals and enzymes, followed by an outer membrane that covers the cortex peptidoglycan (PG) layer which is in turn outside the germ cell wall PG. The cortex in particular, probably with assistance from the coat, participates in establishing and maintaining the reduced water content of the spore core (25%–55% of wet weight, depending on the species) (Riley et al. 2020). Under the germ cell wall is the inner membrane (IM), which in *Bacillus* spores has very low permeability likely due to the very low mobility of IM lipids (Cowan et al. 2004, Sunde et al. 2009). The IM most likely greatly slows entry of damaging chemicals into the core (Kanaan et al. 2022). In addition to the low water content, the core contains high levels, ∼25% of core dry weight, of a 1:1 chelate of dipicolinic acid (DPA) with divalent cations, mainly Ca^2+^ (CaDPA). The spore DNA in the core is also saturated with protective small acid-soluble proteins (SASPs) responsible for spore DNA resistance to desiccation, heat, chemicals, and UV and γ radiation (Christie and Setlow 2020, Nerber et al. 2024). Spores of some species also have an outermost crust layer, others have a loose-fitting exosporium (Fig. 1). *Clostridioides difficile* spores have an exosporium, the structure of which can vary between strains. The latter is believed to contribute to spore resistance, as spores lacking the cysteine rich protein (CdeC) in the exosporium have reduced resistance to lysozyme, ethanol, and heat (Calderón-Romero et al. 2018). Notably, only ∼25% of the more than 80 proteins in the spore coat are conserved between *Bacillus subtilis* and *C. difficile*, which suggests that the spore surface may be an important source of evolutionary adaptations (Christie and Setlow 2020, Riley et al. 2020, Setlow 2019, Lawler et al. 2020). The exosporium of *C. difficile* spores contains three collagen-like glycoproteins, BclA1, BclA2, and BclA3, which are also present in the exosporium of *Bacillus anthracis* spores. Deletion of *bclA1* results in a slight decrease in the ‘virulence’ of *C. difficile* spores, although note that it is only the vegetative cells that are actually virulent. Notably, BclA1 deletion increases both spore germination and adherence, which suggests that changes in the structure of the spore envelope may affect pathogenicity by increasing spore germination and ultimately generation of growing cells (Phetcharaburanin et al. 2014).

## Germination and germinant receptors

Dormant spores have the ability to sense the environment for appropriate conditions for spores to outgrow and lose spore-specific features in a process called germination, a phenomenon that has been best studied in *B. subtilis*. The main events of this process in *B. subtilis* are in order: germinant sensing and recognition, commitment to germinate, release of CaDPA, hydrolysis of cortex PG by spore cortex-lytic enzymes (SCLEs), and finally swelling and full hydration of the spore core. Hydration allows restoration of core protein and IM lipid mobility as well as IM permeability. However, in *C. difficile* spores and those of a few other species, cortex PG hydrolysis precedes CaDPA release (Francis et al. 2015, Bhattacharjee and Sorg 2018). Germinants (these will be discussed in more depth later on) in most *Bacillales* and many *Clostridiales* spores are sensed and likely bound by three protein Ger-type germinant receptors (GRs) in the spore IM, with multiple GRs of different specificities (Warda et al. 2017). Note that recent work indicates that these IM GRs are most likely germinant-gated ion channels (Gao et al. 2023). Once GRs are assembled, they are clustered together in *Bacillales* in one or a few loci on the IM called the germinosome and this is scaffolded by the IM GerD protein which is absent in *Clostridiales* as is, most likely, this type of germinosome; but see below for the possibility of a complex of germination proteins in *C. difficile* spores. The coalescence of the different *Bacillus* GRs in an IM complex increases GR sensitivity of the spores to germinants up to ∼10-fold (Christie and Setlow 2020).

### CspA, CspB, CspC

GRs are also present in the IM of some spore-forming *Clostridiales*. However, *C. difficile* and some related species do not encode orthologs of these GRs but sense germinants and cogerminants through the Csp subtilisin-like serine proteases encoded by genes of the *cspBAC* locus (Francis et al. 2013, Galperin et al. 2022). This operon yields three proteins that are found in the spore cortex, CspA, CspB, and CspC (Baloh et al. 2022). All three of these Csps are vital for *C. difficile* spore germination even though CspA and CspC lack catalytic activity (Kevorkian et al. 2016, Donnelly et al. 2020). CspB and CspA are encoded as a CspBA fusion that undergoes interdomain cleavage during spore formation by YabG, and the resulting proteins are then assembled in the spores’ cortex by an unknown mechanism (Adams et al. 2013, Kevorkian et al. 2016, Shrestha et al. 2019, Baloh et al. 2022). Inactivating or deleting YabG eliminates interdomain processing of CspBA. Probably that leads to misassembly of the coat and exosporium, the formation of spores that are more permeable to lysozyme as well as impaired colonization and host colonization in *C. difficile* (Marini et al. 2023). YabG also processes the cortex destined protein preproSleC into proSleC; mutation of the YabG processing site (R119A) led to YabG shifting its processing to R115 or R112 (Osborne et al. 2024). Both CspA and CspC lack their catalytic portion, and only CspB has a complete catalytic triad composed of aspartate, histidine, and serine residues. Because of that peculiarity CspB is thought to activate the SCLE pro-SleC to its active, cortex-degrading form, SleC, upon CspB activation by CspC through protein-protein interaction. SleC is a cortex lytic enzyme lacking its *N*-terminal pro sequence, and it degrades the cortex, leading to CaDPA release in response to osmotic swelling. Osmotic pressure across the IM is sensed by the IM protein SpoVAC, a mechanosensing protein that together with other SpoVA proteins allows CaDPA release (Velásquez et al. 2014, Francis and Sorg 2016). However, elimination of the catalytic triad of CspB does not fully eliminate germination, indicating that other enzymes are also involved in cortex hydrolysis (Adams et al. 2013, Kevorkian et al. 2016). CspC is thought to be the BA GR, since mutations in *cspC* altered or eliminated the ability of spores to germinate in response to BAs. Such mutations also impaired the ability of the mutant spores to establish infection in animal models, but the compounds that CspC interacts with are unknown (Francis et al. 2013). This evidence is reinforced by the fact that in *Clostridium perfringens*, the orthologue gene *cspC* is also involved in the triggering and regulation of germination, since *cspC* mutants of the SM101 strain were unable to germinate—they were also defective in DPA release and there was no processing of pro-SleC into active SleC (Talukdar and Sarker 2020). CspC is thought to transmit a signal to CspB in a way that is, however, unknown. That inactivation of CspC or SleC inhibited cortex degradation and CaDPA release suggests that CspC is needed for CaDPA release and is consistent with cortex degradation preceding CaDPA release (Francis et al. 2013, Francis and Sorg 2016). CspA is also involved in the sensing of amino acids and calcium cogerminants, and deletion of *cspA* (or loss of YabG) results in a loss of CspC incorporation into spores and thus a germination defect (Kevorkian and Shen 2017, Shrestha et al. 2019).

**Figure 2.**
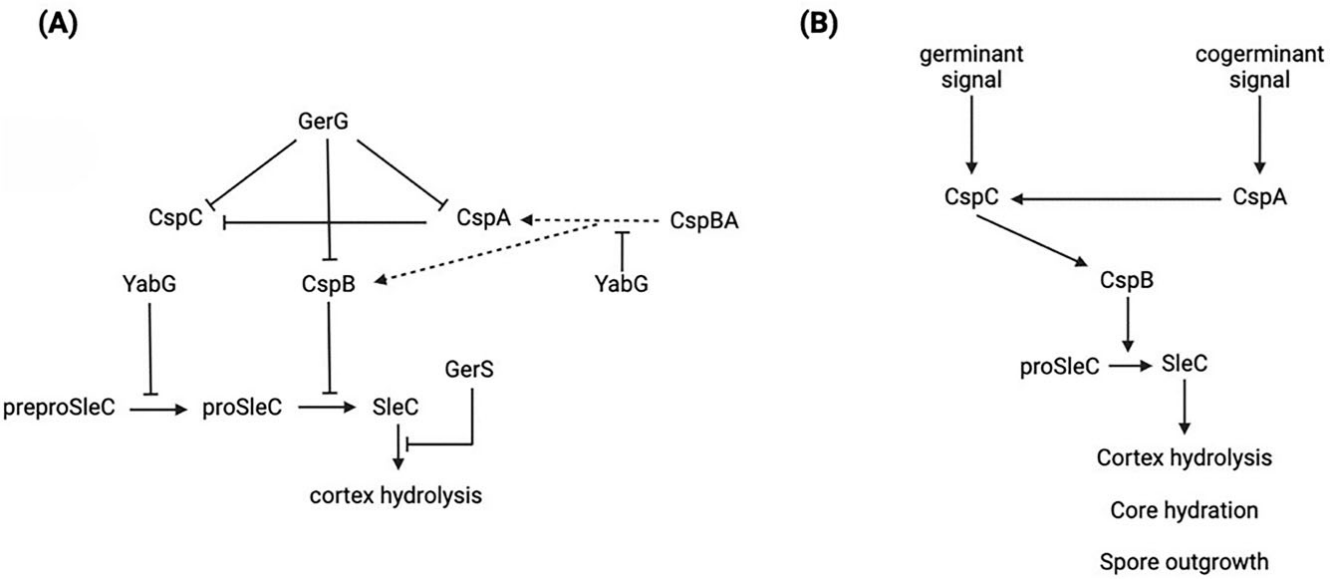
*Clostridioides difficile* spore germination. (A) Functional roles of the most important regulators in *C. difficile* germination, and (B) the series of events that trigger germination in *C. difficile* and their outcome. Arrowheads indicate positive interactions whilst blunt arrows indicate inhibitory interactions. Dotted lines represent presumed interactions. Note that localization of the indicated proteins in *C. difficile* spores has only unequivocally been shown for CspA, B, and C by Baloh et al. (2022), who give clear evidence for a cortex localization of these proteins. YabG is a known coat protein in *Bacillus subtilis* (Takamatsu et al. 2009). Since the indicated known interactions involve processes in the spore cortex, it is a fair assumption that GerG, GerS, YabG, and various forms of SleC are likely to be localized in or close to the cortex in *C. difficile* spores.

Csp proteins are not only essential for germinant sensing, but a proper interaction between them during spore formation is crucial for effective germination. CspA controls levels of CspC and stabilizes it in the developing spore, and spores lacking CspA are defective in germination and have reduced CspC levels (Kevorkian and Shen 2017). However, elevated CspC levels do not lead to more germination in the population assessed, meaning that this protein might have a more complicated role in *C. difficile* germination (Kevorkian et al. 2016). Figure 2 presents a model of *C. difficile* germination regulation pathways taking all these factors into account.

### *gerG, gerS*, and *cd630_32 980* as factors distinctly required for *C. difficile* spore germination

The correct synthesis of the proteins expressed from *gerG, gerS*, and *cd360_32 980* seems to be essential for correct germination of *C. difficile* spores. The expression of these three genes is controlled by the mother cell sigma factor σE, and *gerG* and *gerS* encode proteins that modulate germination. GerG is involved somehow in the insertion of CspA, CspB, and CspC into the mature spore and/or stability of Csps there; gerG deletion decreases the levels of Csps in spores, limiting the detection of germinants and therefore inhibiting cortex hydrolysis (Donnelly et al. 2017). GerS is a lipoprotein, and regulates germination by allosterically activating the CwlD amidase that generates the MAL modification in spore cortex PG, thereby stabilizing the binding of the Zn^2+^ cofactor essential for CwlD activity (Alves Feliciano et al. 2021), and ultimately modulating SleC activity. MAL is a modification specifically recognized by cortex lytic enzymes, ensuring that these lytic enzymes do not degrade the vegetative germ cell wall of spores. Indeed, *gerS* mutant spores have a severe germination defect because the cortex PG lacks the MAL needed for recognition and cleavage by SleC (Fimlaid et al. 2015, Donnelly et al. 2017). While GerG and GerS could be used as targets to develop potential therapies against *C. difficile*, it is notable that at least GerS is conserved throughout the *Peptostreptococcaceae* family (Fimlaid et al. 2015).

Cd630_32 980 encodes an AAA + ATPase that is highly induced during sporulation; this protein likely functions during sporulation since spores lacking this protein are deficient in both calcium and DPA. Nevertheless, this protein seems to be required for taurocholate-glycine germination: mutant spores presenting a deletion of this protein had impaired germination regardless of the glycine concentration (Kochan et al. 2017). Moreover Cd630_32 980 is dispensable for colonization in a murine model of *C. difficile* infection and *ex vivo* germination in mouse ileal contents. Since spores lacking Cd630_32 980 are CaDPA deficient, it has been hypothesized that Cd630_32 980 is involved in transport of CaDPA across the forespore OM (Kochan et al. 2017).

### Germination models

Based on the above-mentioned knowledge, two models of the molecular events in *C. difficile* spore germination have been hypothesized. In the first, the germinant acts as a ‘key’ that binds to the CspC ‘lock’. The activation of CspC then facilitates access to calcium and amino acid cogerminants by a still unknown mechanism. Once CspB binds to endogenous or exogenous calcium it is in its active form and starts transforming proSleC into SleC, which degrades the cortex PG, triggering CaDPA release and core hydration, followed by germinated spore outgrowth (Kochan et al. 2017).

**Figure 3.**
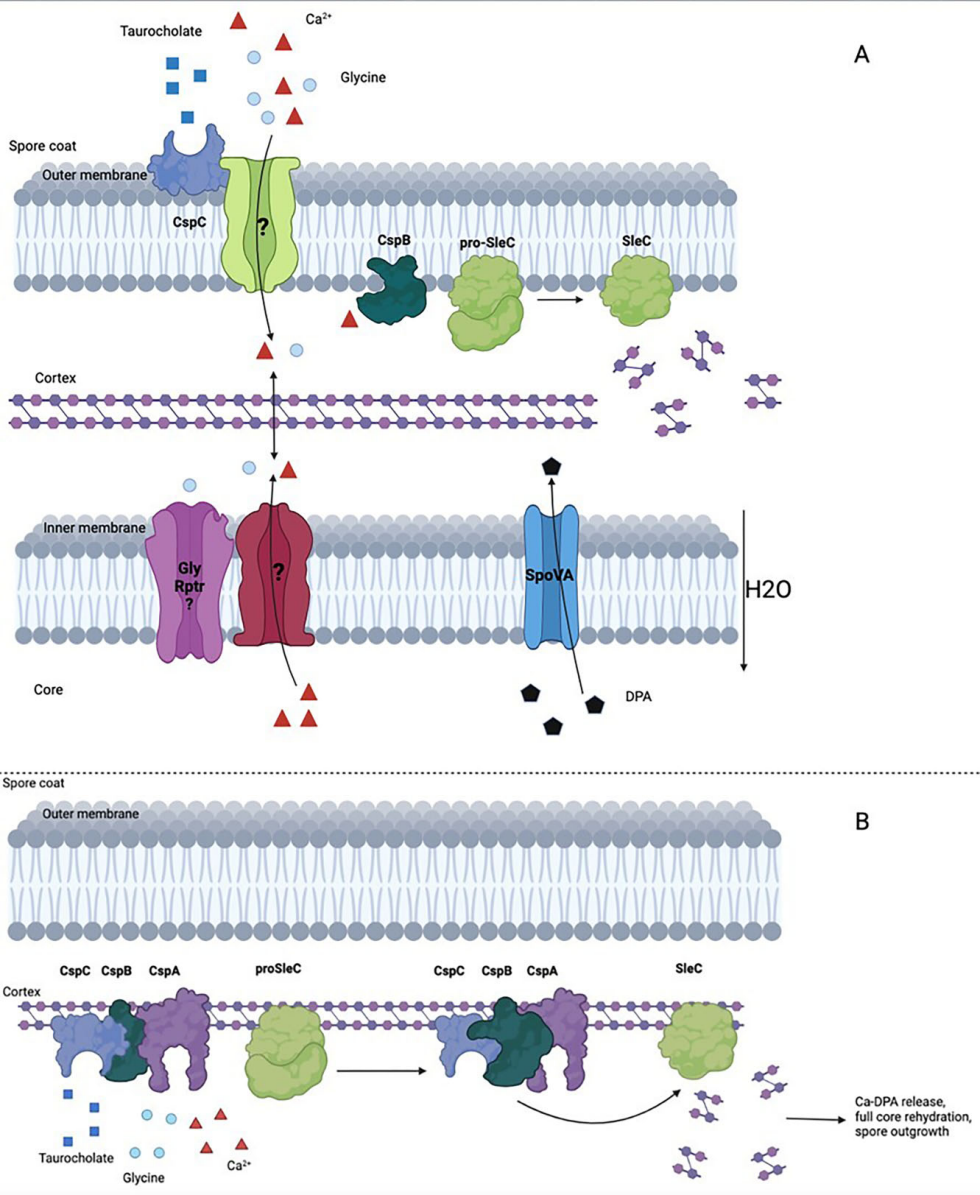
(A) Overview of the *C. difficile* spore germination ‘key and lock’ model. The germinant acts as a ‘key’ to the CspC ‘lock’ facilitating the access to calcium and cogerminants through an unknown mechanism. Once CspB binds to endogenous or exogenous calcium it turns into its active form and starts cleaving proSleC into SleC. SleC can now degrade the cortex PG, triggering CaDPA release and core hydration, followed by germinated spore outgrowth. (B) Overview of the *C. difficile* spore germination ‘germinosome’ model. Once the germinant receptor CspC and the cogerminant receptor CspA are activated, a series of conformational changes is triggered which lead to the activation of CspB. CspB can now process proSleC into SleC which can degrade the cortex, triggering CaDPA release, core hydration, and spore outgrowth.

The second model is the ‘germinosome’ model (Fig. 3) which proposes that CspA, CspB, and CspC are in a complex with proSleC in the cortex. This would facilitate direct interaction between the different proteins of the complex so that CspC and CspA can inhibit the protease activity of CspB by their physical interaction. However, when CspC and CspA bind to a germinant or cogerminants, respectively, they undergo conformational changes. These changes might allow CspB, which is no longer inhibited, to accommodate calcium in its interaction pocket and process proSleC into SleC and trigger cortex degradation and thus germination. CspB calcium binding has not been firmly established though, leaving the above hypothesis for now as speculative. Recent studies (Rohlfing et al. 2019, Shrestha et al. 2019) showed that CspC may not bind germinants directly, but it may interact instead with a GR, still to be identified, as well as the cogerminant receptor to integrate and relay the germination signal to CspC leading to its activation. This may induce a conformational change in the protein that allows it to interact with CspB inducing proteolytic activity and interaction of CspA with CspB. Even though CspA, CspB, and CspC are clearly principal mediators of spore germination, precisely how they initiate the process still has to be elucidated (Rohlfing et al. 2019, Shrestha et al. 2019).

### Germinants

**Figure 4.**
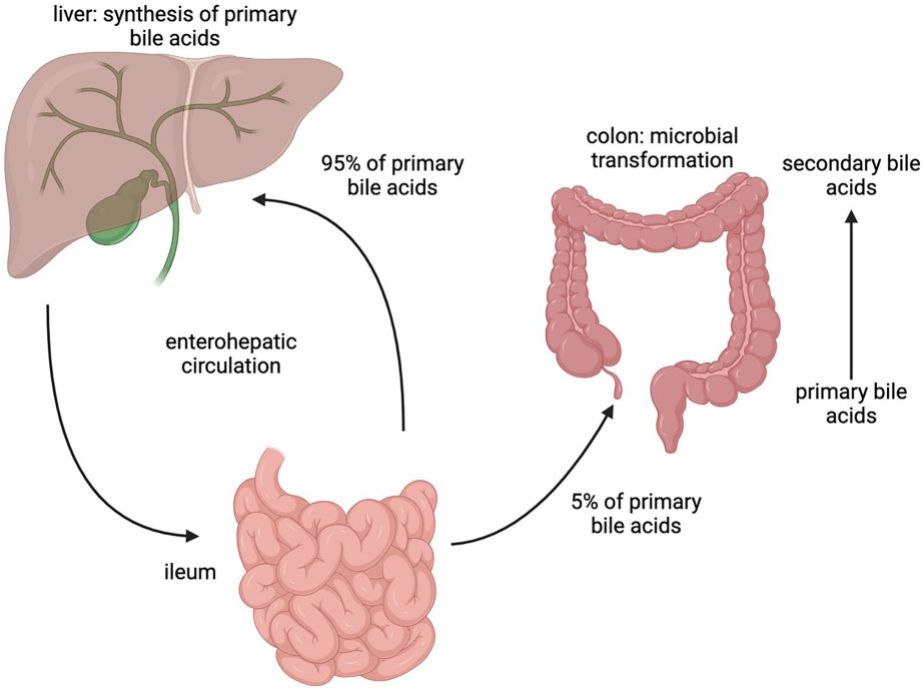
Overview of the enterohepatic circulation of bile acids. The PBAs, synthesized in the liver, are secreted into the small intestine, 95% are reabsorbed at the end of the ileum while the remaining 5% continue to the colon where the microbiota convert them to SBAs by 7α-dehydroxylation.

**Figure 5.**
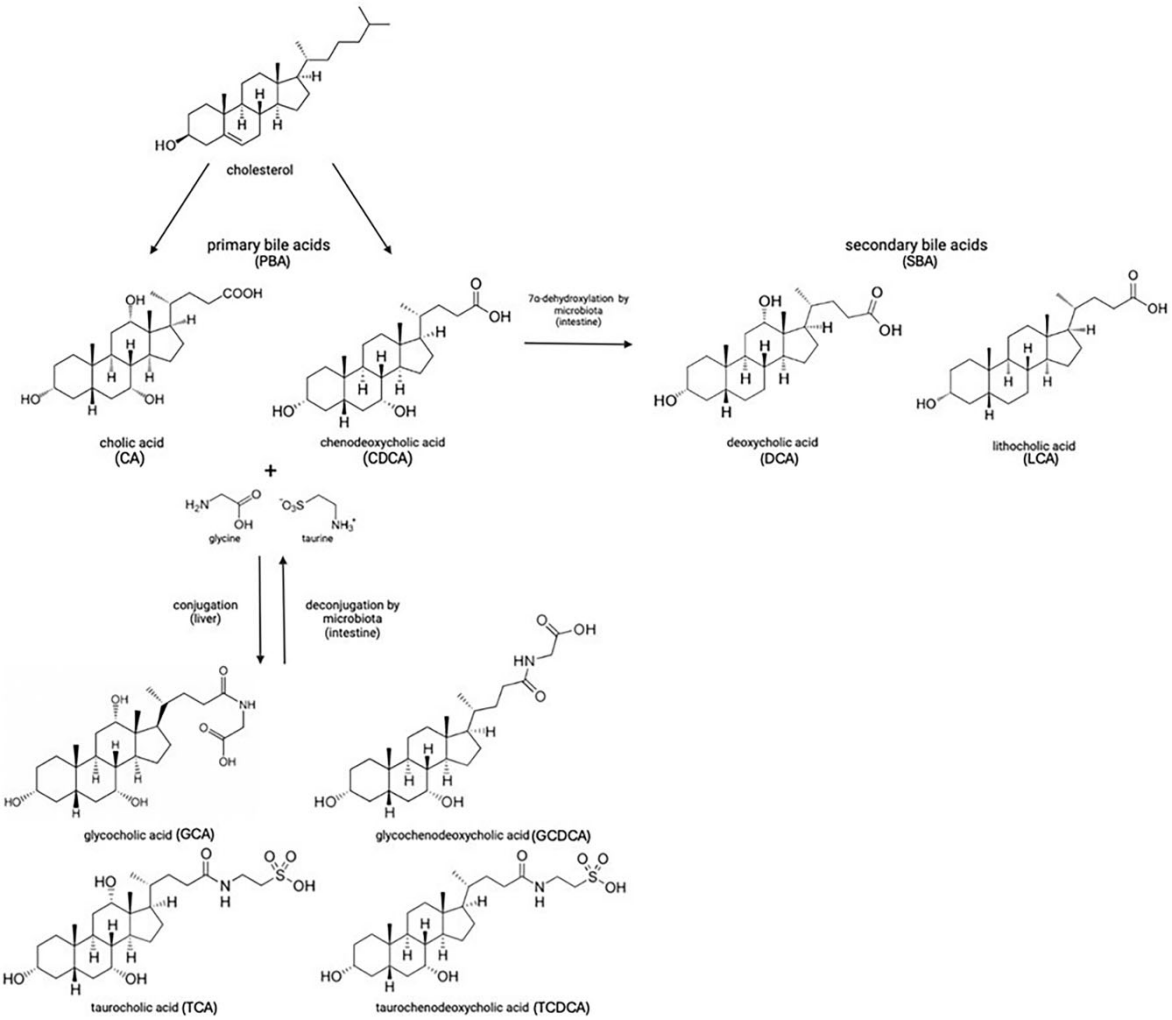
Conversion of PBAs to SBAs: in the liver, bile acid metabolism mainly produces two primary bile acids, cholic acid, and chenodeoxycholic acid; in humans, these are mostly found conjugated with either glycine or taurine. In the intestine, primary bile acids are first deconjugated and then used as substrates for microbial metabolism by the gut microbiota to generate secondary bile acids by 7α-dehydroxylation, including deoxycholic acid and lithocholic acid. Deoxycholate stimulates spore germination but inhibits vegetative growth of *C. difficile* whereas lithocholic acid is insoluble in water and was not analyzed in this regard (Sorg and Sonenshein 2008).

Dormant spores sense the environment for germinants which can trigger spore germination, resulting in vegetative cell growth. The physiological germinants of *Bacillus* and some Clostridial spores are mostly low molecular weight nutrients such as amino acids, sugars or purine nucleosides, but also some ions such as K^+^ or Na^+^. Germination can also be induced by nonphysiological germinants such as: (i) cationic surfactants such as dodecylamine, or (ii) high pressure, generally hundreds of megaPascals; notably, sublethal heat treatment can also increase responsiveness to physiological germinants (Setlow 2019). *Clostridioides difficile* is rare among spore formers, as its spores germinate in response to BAs (Sorg and Sonenshein 2008), although BAs also somewhat increase rates of germination of spores of other *Clostridium* species (Liggins et al. 2011, Browne et al. 2016, Tanaka et al. 2020). BAs are specific compounds made in the liver from cholesterol and secreted into the small intestine where they are crucial for lipid absorption and metabolism. These compounds are then reabsorbed in the lower region of the small intestine and returned to the liver. BAs are also involved in several important signaling mechanisms. For example, they are ligands for the ‘membrane bile acid receptors’, TGR5, a G protein-coupled receptor involved in glucose homeostasis and the ‘nuclear bile-acid receptor’, also named FXR-α which regulates the enterohepatic recycling and biosynthesis of BAs (Wang et al. 1999, Maruyama et al. 2002). The two most important primary bile acids (PBAs) are cholic acid (CA) and chenodeoxycholic acid. In humans, most PBAs are conjugated with either taurine or glycine in a 1:3 ratio (Chiang 2013) forming the conjugated PBAs taurocholic acid (TCA), glycocholic acid (GCA), taurochenodeoxycolic acid, and glycochenodeoxycolic acid. Most PBAs secreted into the gut are reabsorbed and transported to the liver; nevertheless, some avoid reabsorption and pass to the large intestine (Dawson and Karpen 2015). Here, action of BA hydrolases located on the cell surface of many different bacteria, converts conjugated PBAs (a large fraction of the total PBAs) into their deconjugated form (Fleishman and Kumar 2024). Finally, a subset of the colonic microbiome will take up and 7α-dehydroxylate the PBAs to form secondary bile acids (SBAs) (Figs. 4 and 5) (Ridlon et al. 2006). Some of the most important SBAs in the human large intestine are deoxycholic acid (DCA), lithocolic acid (LCA) isodeoxycholic acid (iDCA), and isolithocholic acid (Fleishman and Kumar 2024). Of the PBAs, TCA, GCA, and CA but not CDCA or ursodeoxycholic acid, the 7 ß epimer of CDCA, support outgrowth of *C. difficile* vegetative cells from spores. CDCA in fact inhibits spore germination (Sorg and Sonenshein 2008). Conversely while SBAs may inhibit *C. difficile* spore germination or, as is the case for DCA, promote it, *C. difficile* vegetative cells grow poorly if at all in their presence. A healthy gut has a microbiome that produces elevated concentrations of SBAs preventing *C. difficile* vegetative growth while, by reducing the biodiversity of the gut microbiome, the amount of germinants (conjugated PBAs) rises because of the lower conversion rate of PBAs into SBAs which is usually performed by a healthy microbiota (Diederen et al. 2020). Of note, there is compelling recent evidence from Girinathan et al. (2021) using gnotobiotic mice, indicating that metabolic competition in a healthy gut rather than specific BA action can play a major role in the protection from *C. difficile* cell overgrowth. Indeed, using mice, Aguirre et al. (2021) showed evidence supporting the notion that while SBAs presence may be a good biomarker for a *C. difficile* resistant intestinal environment, mechanistically they may be dispensable (see also Aguirre and Sorg 2022).

In any case, since an intestinal tract that has more PBAs correlates with a lower bacterial diversity, it is potentially a more suitable environment for *C. difficile* to take advantage of. Thus, it could be hypothesized that *C. difficile* evolved GRs that had higher affinity for PBAs than SBAs, as the latter corresponded to a healthier, less inviting, microbiome. For example, it was found that *Clostridium scindens*, a commensal bacterium capable of 7α-dehydroxylating BAs could enhance the resistance to CDI by generating SBAs capable of inhibiting *C. difficile* vegetative growth (Buffie et al. 2015). Studies on mice models showed that treatment of CDI by administration of *Bacteroides thetaiotaomicron* or fecal matter transplantation was associated with restoration of the intestinal microbiota and, most importantly, reversed changes in BAs composition associated with *C. difficile* infection. Indeed, fecal analyses from the same study found that ratios of promoting/inhibiting BAs (where promoting BAs promote the proliferation of *C. difficile* and inhibitory BAs blocked vegetative growth) were significantly decreased and even lower than the control group. This study also suggested a new way of treating CDI using probiotics able to increase the SBA/PBA ratio instead of antibiotics (Li et al. 2021). Another study carried out on a cohort of 56 patients showed that gut BA metabolism dynamics differ in primary CDI patients between those who develop recurrence compared to those who do not. Two of the fecal BAs with the most marked difference between groups were DCA and LCA (both SBAs) and their glycine-conjugated forms, with significantly higher levels in nonrecurring patients. Conversely patients experiencing recurrence had higher stool levels of PBA derivatives like sulfated and/or amide-conjugated ursodeoxycholic acid, or those similar to the 5β-cholanic acid (Mullish et al. 2022). A recent study also linked BA metabolism to *C. difficile* pathogenesis: in mice, challenged with 10^5^ spores, the addition of a bile salt hydrolase cocktail significantly inhibited *C. difficile* spore germination in the small intestine of all mice and suppressed growth. Furthermore, metabolomic analyses of stool BAs indicated that the bile salts hydrolase cocktail remained active and generated an increase in deconjugated BAs (significantly more inhibitory than their conjugated version), reshaping the BAs pool (Foley et al. 2023).

Interestingly, a study of colonic bacteria conducted on a cohort of Japanese centenarians, revealed that they present some centenarian-specific microbial taxas; one of the most enriched species was *C. scindens* which, as previously mentioned, can convert PBAs into SBAs. The distinct gut microbiome of centenarians leads to the production of SBAs unique to this age group, such as various isoforms of lithocholic acid, produced by a subset of enzymes found in a few commensals including *C. scindens* and *Clostridium hylemonae*. It might be that these SBAs are involved in the decreased susceptibility of elderly people to enteropathogenic infections and promote intestinal homeostasis perhaps by reducing growth of various microbes (Sato et al. 2021). Those data suggest that PBAs might be used as biomarkers for people more sensitive to CDI and its recurrence. In general, BA germinants and inhibitors are structurally similar, it is the presence or absence of the 12α-hydroxyl group that makes a compound a germination promoter or suppressor. It is also important to note that the effectiveness of germinants and inhibitors can vary widely across clinically relevant strains of *C. difficile* (Sorg and Sonenshein 2010). Although necessary, BAs alone are not sufficient to trigger germination in the small intestine, as amino acid cogerminants are also essential. Different amino acids give different germination efficiencies (Shrestha and Sorg 2018); glycine is the most effective cogerminant but L-alanine, L-glutamine, and taurine have cogerminant properties. In general, amino acids with branched side chains have the least cogerminant properties and, surprisingly, amino acids that regulate important physiological processes (L-isoleucine, L-leucine, L-valine) are not cogerminants. In strains with alanine racemase (Alr2) that can interconvert L-alanine and D-alanine, D-alanine can also act as a cogerminant (Shrestha and Sorg 2018). Alr2 also interconverts L- and D-serine and both these amino acids can act as cogerminants, and histidine was also found to enhance taurocholic- and glycine-mediated germination. The action of amino acids as cogerminants is highly temperature dependent; many amino acids only act as cogerminants at 37°C (lysine and serine), but not at 25°C, the latter a temperature at which many experiments are conducted when mimicking more environmental conditions (Shrestha et al. 2017, Shrestha and Sorg 2018).

Some work has also suggested that Ca^2+^ is indispensable for *C. difficile* spore germination (Kochan et al. 2017), however, more recent work suggests this is not necessarily true (Ribis et al. 2023). Some work suggested Ca^2+^ might have a synergistic effect with amino acid cogerminants, but since it plays an important role in the proper functioning of CspA, CspB, CspC, and the cortex hydrolase or for a still undefined calcium-dependent enzyme needed for signal transduction, it might not function as a cogerminant but rather as a co-factor for *C. difficile* spore germination. *Ex vivo* assays in a murine model also showed that spore germination did not take place in a Ca^2+^ depleted ileum, suggesting an interesting way to control germination by modulating intestinal Ca^2+^ levels (Kochan et al. 2017).

### The *C. difficile* life cycle inside and outside the gut

Besides occupying an ecological niche in for instance soil, the life cycle of *C. difficile* may have adapted to the coprophagic behavior that many animals like leporids, insects, rodents, canids, large herbivores, and nonhuman primates have in the wild (Spitzer et al. 2023). Perhaps adapting to survive in the drastic change of environment between the inside and the outside of the digestive tract gives *C. difficile* a way to easily spread through the feces within the same species or to others (see graphical abstract).

Evidence coming from world-wide studies supports the hypothesis that mammal fecal matter, especially human, might be the vector for *C. difficile* to spread in the environment. In particular, a study in western Australia (Chisholm et al. 2023) analyzing waste-water treatment plants found that *C. difficile* spores are present in most of the raw sewage influent (90.5%), 48.1% of the treated influent, 40% of the reclaimed irrigation water and 100% of untreated biosolid. Notably, over half of the isolated spores were toxigenic, including the hypervirulent RT078 strain. This study revealed that toxigenic *C. difficile* was released into the environment. Another study (Xu et al. 2014) also reported a high prevalence of *C. difficile* in raw sludge (92%), anaerobically digested sludge (96%), biosolids (73%), and sediments (39%) in Southern Ontario watersheds. Moreover, research conducted in western Australia found *C. difficile* spores in two thirds of home garden samples, 38% of the isolates being toxigenic (Shivaperumal et al. 2020). Even more intriguingly, an overlap was found between the genotypes isolated from the environment and those isolated from the hospital patients in the same area.

Humans are not the only host for *C. difficile*, pigs and horses can also be affected. Spores inoculated into pig feces were able to survive for over 30 days in a pig feces-based manure compost (Usui et al. 2017). A total of 11 strains, 82% of them being toxigenic, were isolated from 5 of 14 final products of manure compost products. Therefore, the use of composted manure on land poses a possible risk of *C. difficile* spread. *Clostridioides difficile* causes diarrhea in horses, with the main sources of infection being equine and other animal feces, contaminated soil, and animal hospitals (Diab et al. 2013, Usui et al. 2017). All these data support the idea of *C. difficile* being evolved to spread in the environment in the form of spores which then germinate in the intestinal tracts of humans and other mammals and reproduce.

Evolution has equipped *C. difficile* spores with a set of GRs that trigger germination only in the presence of a combination of compounds present in the very specific environment of the mammalian large intestine, and CspA and CspC are essential for correct germination (Francis et al. 2013). It has been established that CspC is essential for correct germination in an animal model and mutations in *cspC* alter the specificity of germinant recognition or abrogate the ability of *C. difficile* spores to germinate in response to BAs (Francis et al. 2013). Moreover, while the loss of CspB catalytic activity led to a 20-fold germination reduction, mutants in the pseudoprotease domain of the cogerminant receptor CspA led to a 100-fold defect in spore germination. CspA also has an important role in regulation of CspC levels during spore maturation, probably acting as a scaffold protein; this is consistent with proper sensing of all needed inputs being essential for efficient spore germination (Shrestha et al. 2019).

*Clostridioides difficile* is present worldwide in a large part of the population (Czepiel et al. 2019) in its vegetative form but its presence does not always lead to the development of an illness. *Clostridioides difficile* is an opportunistic species, adapted to take advantage usually of individuals having a depleted gut microbiota, unable to efficiently deconjugate and 7α-dehydroxylate PBAs into SBAs, thereby enabling *C. difficile* to sense germinants and then outgrow into the vegetative form. Nevertheless, its presence does not always correlate with the development of symptoms. CDI usually affects people following broad spectrum antibiotic treatment that reduces intestinal microbiome diversity so that they are not able to metabolize PBAs into SBAs due to the lack of bacterial BA dehydroxylating enzymes. A higher concentration of primary PBAs in stool is a biomarker for CDI and its potential recurrence, since PBA levels were significantly elevated in recurrent CDI patients compared to control and first time CDI patients; using random forest regression recurrent CDI and first time CDI patients were distinguished 84.2% of the time (Mullish et al. 2022). The stool deoxycholic and glycoursodeoxycholic acid ratio was the single best predictor. Moreover, the presence of the SBAs taurodeoxycholic and glycodeoxycholic acid in the plasma were the best single indicators of disease status when considering all the BAs as a factor (Allegretti et al. 2016). There is also a relation between low levels of SBAs in the gut and CDI recurrence (Allegretti et al. 2016, Mullish et al. 2022). Correlation between high PBA concentrations and symptomatic carriers of *C. difficile* was also found in fecal samples. Not only does the level of BAs differ in CDI patients and asymptomatic carriers, but the relative abundance of the spores is significantly different: one study revealed that healthy carriers had a stool density of 3.0 × 10^4^ colony forming units per gram (CFU/g) while CDI patients had 4.0 × 10^6^ CFU/g (Naaber et al. 2011). These insights may help identify those at highest risk for recurrent disease and support the development of new treatments that target bile salt metabolism. Unfortunately, not enough data are available to know whether there is an association between faulty hepatic uptake of PBA and CDI. Nevertheless, all available data suggest an evolutionary adaptation by *C. difficile* for germination in unhealthier guts where there is a higher chance for this opportunistic bacterium to proliferate, although spore germination does not always lead to disease. Finally, it is important to note that in infants, the levels of CA and CDCA are quite high until the third year of life, as is discussed in Cheng et al. (2019). Note that CDCA is an inhibitor of *C. difficile* spore germination, while CA supports germination and outgrowth but is not the optimal germinant. It might be that this composition along with the right metabolic environment (see Aguirre et al. 2021) protects the infant gut from CDI. If anything, given that levels of *C. difficile* spores are quite high in infants (Shirley et al. 2023), this may well be an important human reservoir of *C. difficile*.

## Conclusion

This review attempts to summarize current knowledge on *C. difficile* spore germination, it also discusses evidence of the adaptation of this bacterium to a life cycle carried out inside and outside the human digestive tract.

The evidence presented suggests that *C. difficile* exploits sporulation to survive and spread into the environment and adapt to preferably germinate in the digestive tracts of individuals with disturbed microbiomes.

*Clostridioides difficile* spores are spread in the environment not only from human hosts but also because they can colonize other mammals and by technologies such as irrigation and wastewater recycling. The realization that *C. difficile* is exquisitely adapted to a life cycle that takes place both inside and outside the gut can help in combating infections with this pathogen.
